# Integrating Case Management in Cystic Fibrosis Units: A Key to Enhancing Patient-Centered Care

**DOI:** 10.3390/healthcare13090965

**Published:** 2025-04-22

**Authors:** Alessandra Russo Krauss, Andrea Lastrucci, Valentina Petrini, Nicola Gualtieri, Renzo Ricci, Matteo Tomaiuolo, Daniele Giansanti, Alessandro Bartoloni, Silvia Bresci

**Affiliations:** 1 Department of Allied Health Professions, Azienda Ospedaliero-Universitaria Careggi, 50134 Florence, Italy; 2 Clinical Operating Unit of the Organizations, Careggi University Hospital, Regional Reference Center on Relational Criticalities (RCRC), 50141 Florence, Italy; 3 Hospital Management Unit, Careggi University Hospital, 50134 Florence, Italy; 4 Centre TISP, Istituto Superiore di Sanità, 00161 Roma, Italy; 5 Department of Experimental and Clinical Medicine, University of Florence, 50134 Florence, Italy; 6 Infectious and Tropical Diseases Unit, Careggi University Hospital, 50134 Florence, Italy

**Keywords:** case management, cystic fibrosis, multidisciplinary care, telemedicine, care coordination

## Abstract

**Introduction**: Cystic fibrosis (CF) is a chronic genetic disease affecting the respiratory and digestive systems. Multidisciplinary care is vital for managing CF’s complex complications. This study investigates the potential role of a Case Manager (CM) in improving care coordination and patient outcomes at the CF Unit of Careggi University Hospital. **Methods**: A survey among 34 CF Unit healthcare professionals assessed the perceptions of integrating a CM. The survey included demographic questions and 12 Likert scale items on the CM’s role in care continuity, team collaboration, and treatment adherence. Responses were collected anonymously and analyzed using descriptive statistics. **Results**: The response rate was 100%, with strong support for the CM role, averaging 4.5/5 across Likert scales. Respondents highlighted the CM’s value in coordinating care, managing time-sensitive tasks, and improving communication with external care providers. Telemedicine was positively rated, particularly for reducing hospital visits and supporting the remote monitoring of CF patients treated. **Discussion**: Findings indicate that integrating a CM could enhance multidisciplinary CF care by improving communication and treatment adherence. Challenges, including team readiness and training, were noted. Future studies will focus on patient satisfaction and clinical outcomes following the integration of CM, with special attention to the role of telemedicine in CF care.

## 1. Introduction

Cystic fibrosis (CF) is a chronic disease that causes significant damage to the respiratory and digestive systems. It is the most common genetic disease among Caucasians. The disease is caused by mutations in the CFTR gene (cystic fibrosis transmembrane conductance regulator) [1]. The epidemiology of the disease has changed significantly in recent decades, mainly due to advances in prenatal screening, developments in CF care and organ transplantation [2], and the introduction of new protein-modulating therapies. As a result, CF affects more adult than pediatric patients [3]. CF patients experience a range of clinical manifestations, including progressive pulmonary obstruction, sinusitis, pancreatic and liver dysfunction, malabsorption, and malnutrition. Due to these characteristics, a multidisciplinary approach is essential for effective disease management [4]. In addition, the disease progresses with age and the complexity of care increases as patients age. Pulmonary complications such as hemoptysis, pneumothorax, exacerbations, and respiratory failure, as well as non-pulmonary complications such as diabetes, osteoporosis, anxiety and depression, kidney disease, hearing loss, cardiovascular disease, male infertility, difficulties for women to carrying a pregnancy to term, and an increased incidence of certain cancers compared to the general population are more common in adult patients. These conditions are in addition to the usual age-related diseases also found in the general population [2]. Given this complexity, an effective approach to managing CF requires not only multidisciplinary care but also the structured coordination of services. In this context, case management, defined as “a collaborative process of assessment, planning, facilitation, care coordination, evaluation, and advocacy for options and services to meet an individual’s and family’s comprehensive health needs through communication and available resources to promote quality, cost-effective outcomes” [5] plays a central role in most integrated and person-centered care services. In these settings, the provision of proactive and preventive care, oversight of care coordination, and team collaboration are paramount [6]. Particularly in multidisciplinary and multiprofessional teams, case management enables individualized care for each case in order to personalize the care pathway and thereby increase efficiency, including cost-effectiveness [7]. This approach is especially crucial for populations with chronic diseases, who often require complex, ongoing, patient-centered, and long-term care that extends across multiple healthcare settings [8]. Internationally, case management has been shown to be an effective, efficient, and cost-conscious strategy for coordinating care in chronic disease management [9]. This approach is often integrated into disease management programs, which are designed to manage chronic conditions or high-cost health problems with maximum efficiency. The primary goal is to prevent or minimize exacerbations that could lead to costly interventions such as hospital admissions [10].

A retrospective observational cohort study [11] analyzed the effects of case management in adults with multimorbidity before and after its introduction. The study emphasized the need for complex, multifactorial interventions that emphasize both logistical and educational aspects to reduce healthcare resource use. By integrating community and hospital services, the researchers evaluated the impact of planned follow-up calls by case managers (CMs). Compared to previous research, the significance of this study lies in the results, which show that both unplanned hospital admissions and hospital stays were reduced by almost 50%. The CM establishes a trusting relationship, with both the healthcare team and the patient, and develops a prevention plan based on clinical guidelines and tailored to the patient’s specific socio-health conditions. Ongoing contact with the patient is established and maintained by telephone calls and emails or, if necessary, via telemedicine [10]. The World Health Organization (WHO) defines telemedicine as “the delivery of healthcare services where patients and providers are separated by distance. Telemedicine uses information and communication technology (ICT) to exchange information for the diagnosis and treatment of diseases and injuries, for research and evaluation and for the training of healthcare professionals” [12]. In addition to overseeing telemedicine, the CM plays a crucial role in patient education. By educating patients and their families about disease management, therapy adherence, and self-monitoring techniques, the CM empowers individuals to take an active role in their own care. This proactive approach not only improves health outcomes but also reduces the likelihood of avoidable hospital admissions and disease complications [11].

### 1.1. AIM

The aim of this study is to assess the potential integration and impact of the Case Manager (CM) within the multidisciplinary team of the Cystic Fibrosis (CF) Unit at Careggi University Hospital, which is working to establish a dedicated service for adult CF care and facilitate the transition from pediatric to adult services. The study specifically seeks to achieve the following:

Explore how healthcare professionals in the CF Unit perceive the CM’s role in improving care coordination, interdisciplinary collaboration, and continuity of care for adult patients.

Evaluate the feasibility of implementing the CM role, particularly by health assistants, to enhance treatment adherence and support the use of telemedicine for remote patient management.

#### Sub-Aim

This investigation is based on feedback collected through an anonymous electronic survey administered to the CF Unit’s multidisciplinary team, consisting of infectious disease specialists, medical residents, a pneumologist, physiotherapists, dietitians, nurses, health assistants, and a psychologist. The survey aims to gather insights into their understanding of the CM role and its potential contribution to enhancing patient care and team collaboration.

## 2. Materials and Methods

The CF Unit of Careggi University Hospital has planned to develop a dedicated service for the care of CF patients in adulthood during the transition phase from the Pediatric Cystic Fibrosis Unit of the Meyer University Hospital. The CF Unit is composed of multidisciplinary and multiprofessional team consisting of the following:

- 6 infectious disease specialists;
- 15 medical residents;
- 1 pneumologist;
- 4 physiotherapists;
- 2 dietitian;
- 3 nurses;
- 2 health assistants;
- 1 psychologist.

These are the healthcare professionals who work in clinical practice within the CF Unit, but on a daily basis, other healthcare professionals from different medical specialties interact with the CF Unit and its staff in the treatment and management of CF patients. The main site for outpatient clinical activities is a hospital department called ‘Mario Fiori’, located within the Careggi University Hospital. In a multidisciplinary meeting in July 2024, a brief introduction was given to the role and tasks of the CM and the possible implementation of this position within the CF Unit. Following this meeting, an invitation to complete an anonymous electronic questionnaire, reported in the Supplementary Material, was sent to all healthcare professionals working in the CF Unit of Careggi University Hospital to obtain their opinion on the possible introduction of a CM. The 34 professionals included in the study are officially assigned to the CF Unit and are directly involved in the comprehensive management of patients. Other healthcare professionals from different medical specialties interact with the CF Unit on a case-by-case basis, contributing to the multidisciplinary care approach, but their interactions are not structured enough to justify their inclusion, considering this study is a pilot project focused on professionals who have continuous and direct involvement in patient management. Anonymity and confidentiality were guaranteed. Participants were informed of the purpose of the study and voluntarily agreed to participate by answering the specific statements included at the beginning of the questionnaire.

The electronic questionnaire is a virtual tool used to obtain structured feedback from healthcare professionals in the CF Unit. An anonymous survey was carried out using a modern Computer Aided Web Interviewing (CAWI) tool. Microsoft Forms was used to develop this CAWI tool, which was specifically chosen for its seamless integration with Office 365 (version 2024). These strategic choices regarding participants and data collection tools were carefully made to ensure the efficiency, reliability, and ethical integrity of the research process. The CAWI instrument was used in a peer-to-peer process to ensure respondent anonymity. The survey consisted of 16 questions, including 4 single-choice questions and 12 questions that utilized a Likert scale with a 5-point rating. The first four questions collected sociodemographic information, such as gender, age, professional profile, and number of years in the CF Unit. The remaining questions were Likert scale questions focusing on the CM profile and designed on evidence from a qualitative study which aimed to analyze the possible expressions of the Case Manager role and the characteristics of the professionals who embody this role [13]. Rather than serving as a standardized psychometric tool, the questionnaire was developed as an investigative instrument to translate qualitative findings into structured data collection.

The items included in the questionnaire were developed through expert consultation, with the support of the psychology team of the Regional Center for Critical Relationships at Careggi University Hospital.

The key elements addressed in the Likert scale questions are summarized in Table 1.

The methodology included the definition of a roadmap in which the results of the questionnaires completed by healthcare professionals played a central role. This project was organized and coordinated by the Department of Healthcare Professionals and the Infectious and Tropical Diseases Unit, which manages the CF Unit at Careggi University Hospital. The study was aimed at healthcare professionals working in the CF Unit, and all individuals participated as part of their professional responsibilities, with responses being voluntary and anonymous.

In analyzing the responses to the questionnaire, percentages and ratios were used to describe ordinal and nominal data, respectively, while means and standard deviations were used for continuous variables. Individual ratings and Likert scale responses were scored on a scale from a maximum of 5 to a minimum of 1. An average score of more than 3.0 (calculated as (1 + 5)/2) indicated a positive evaluation, with higher scores close to 5 indicating more positive responses. A score below 3.0, on the other hand, indicated a negative evaluation, with scores close to 1 indicating a more critical attitude.

## 3. Results

### 3.1. Descriptive Analysis of the Sample

This survey provided structured feedback on the awareness of the CM professional profile and assessed the potential value of this role within the multidisciplinary team of the CF Unit. Overall, a total of 34 healthcare professionals participated in the survey, achieving a 100% response rate (*n* = 34). The CAWI (Computer-Assisted Web Interviewing) tool was used, not only as a method for gathering structured feedback but also as a means of conducting a virtual focus group for a feasibility project. The participants in the CAWI survey reflected the healthcare professionals involved and interested in the feasibility project.

**Figure 1:**
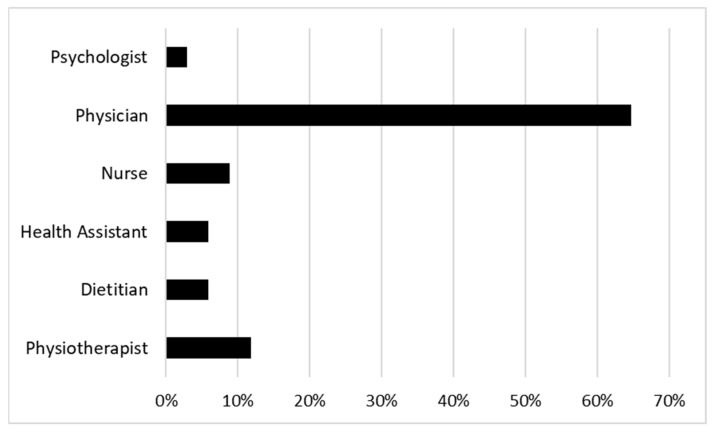
Distribution of the sample by professional profiles.

Table 2 and Figure 1 provide detailed information on the healthcare professionals who participated in the survey. Table 2 provides an overview of the entire sample, while Figure 1 focuses specifically on the distribution of professional profiles among the respondents.

The gender distribution within the study sample shows a predominance of female participants, accounting for 61.76% (*n* = 21) of the total, compared to 38.24% (*n* = 13) for male participants.

This distribution aligns with the overall representation of personnel participating in the virtual focus group and reflects the demographic makeup of those interested in the project. Such consistency indicates that the feedback collected is representative of the broader team, ensuring that the insights gained are reflective of the perspectives and experiences of the healthcare professionals involved in the CF Unit.

The majority of the participants were physicians (*n* = 22, 64.71%), with the second most represented category being physiotherapists (*n* = 4, 11.76%). The remaining professional categories were represented in the sample as follows: nurses (*n* = 3, 8.82%), dietitians (*n* = 2, 5.88%), health assistants (*n* = 2, 5.88%), and psychologists (*n* = 1, 2.94%).

As reported in Table 3, the majority of healthcare professionals in the study sample (*n* = 21, 61.77%) have been employed at the Careggi University Hospital from 2 to 5 years. Smaller percentages are represented by those with less than 2 years of service (*n* = 2, 5.88%), 6 to 10 years (*n* = 2, 5.88%), 11 to 15 years (*n* = 3, 8.82%), 16 to 20 years (*n* = 2, 5.88%), and over 20 years of service (*n* = 4, 11.77%).

Table 4 shows the distribution of the sample based on the years of service at CF Unit. The majority of healthcare professionals (*n* = 22, 64.71%) have less than 2 years of service in the CF Unit. Those from 2 to 5 years of service represent 20.59% (*n* = 7), while smaller proportions have 6–10 years (*n* = 3, 8.82%) and 11 to 15 years (*n* = 2, 5.88%) of experience.

### 3.2. Feedback and Findings from Likert-Scale Sentences

**Figure 2:**
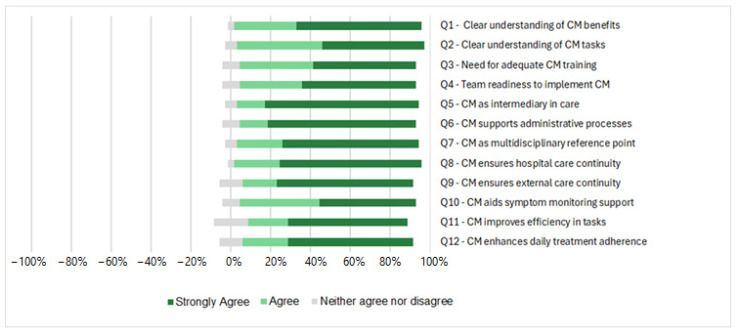
Representation scheme of the answers to the Likert sentences.

The butterfly diagram reported in Figure 2 shows the responses to 12 statements regarding the integration of a CM into the healthcare team of the CF Unit at Careggi University Hospital. The data were collected using a 5-point Likert scale, with 1 being “strongly disagree” and 5 being “strongly agree”. As shown in Figure 2, all the responses in the butterfly diagram demonstrate a significant level of acceptance, as evidenced by the low tail extending below zero across all categories. The majority of respondents chose “agree” or “strongly agree” for all questions. The average ± standard deviation (SD) value of all statements was 4.5 ± 0.1.

Overall, the results obtained from the Likert responses reflect a generally positive attitude towards the integration of a CM, although there is some variability in the responses in certain areas, such as team readiness and communication with external healthcare structures dealing with the time-sensitive task, as follows:

- Team readiness: The need for the adequate training of staff emerges as a key factor for the successful integration of the CM role. When asked, “I think our team is ready to introduce the CM role—Q4”, the majority of respondents answered “strongly agree” (*n* = 20) with a mean score of 4.5 (SD ± 0.7), although a few respondents remained neutral (*n* = 3).
- Role in external continuity of care: A similar pattern was observed when respondents were asked about the role of the CM in ensuring continuity of care outside the hospital. Most respondents fully agreed with the statement “I think the CM can ensure continuity of care by facilitating communication with external structures—Q9” (*n* = 24) with a mean score of 4.6 (SD ± 0.7), although some respondents remained neutral and selected “neither agree nor disagree” (*n* = 4).
- Managing time-critical tasks: Responses also showed general agreement that a CM could play a crucial role in managing time-critical tasks. The majority fully agreed with the statement “I think the CM could make a decisive contribution to ensuring that tasks” are completed on time—Q11” (*n* = 21), with an average score of 4.4 SD ± 0.8), although a few respondents chose the neutral option “neither agree nor disagree” (*n* = 6).

## 4. Discussion

Involving the CM in the management of complex chronic conditions is a critical strategy for optimizing patient care and improving health outcomes. Patients with complex chronic conditions, such as CF, require ongoing multidisciplinary coordination to effectively manage their multiple therapeutic and diagnostic needs. In this context, the CM plays a central role in facilitating collaboration within the care team to ensure personalized, high-quality care [14]. CMs play several key roles in the management of complex chronic diseases. As the central coordinator within a multidisciplinary team, they facilitate communication between different healthcare professionals—such as physicians, nurses, physiotherapists, and dietitians—and the patient. This coordination is particularly important for conditions such as CF, where care management requires adherence to strict schedules for diagnostic tests, specialist consultations, and follow-up care [13]. One of the main tasks of the CM is to optimize the patient’s treatment pathway. This includes scheduling appointments, ensuring diagnostic tests are completed in a timely manner, and overseeing that results are available to the care team for immediate clinical assessment. By completing back-office tasks, the CM helps reduce delays and inefficiencies, improving the overall patient experience and enabling the treatment team to work more efficiently. In addition, the CM plays an important role in supporting treatment adherence, especially with regard to regular testing [15]. According to Zitter, “disease management is a comprehensive integrated approach to care and reimbursement based fundamentally on the natural course of a disease, with treatment designed to address the illness with maximum effectiveness and efficiency” [16]. A key role of the CM within these programs is to provide patient education [10]. The professional profile of the healthcare assistant, as outlined in Italian Ministerial Decree 69/2007, includes several competencies that are closely related to the CM, especially in the care of chronic patients. Healthcare assistants are tasked with health promotion, education, and the coordination of disease prevention and control efforts. These skills align well with the role of the CM, who not only coordinates care but also works to educate patients, improve their understanding of disease management, and promote adherence to treatment plans. According to Ministerial Decree 77/2022 (Annex I, paragraph III), the healthcare professionals who can assume the role of CM in the various phases of patient care, depending on the presence of specific care needs, include midwives and professionals in the fields of technical healthcare and rehabilitation. In Italy, the most common CMs are educators, nurses, psychologists, social workers, or other healthcare professionals with management and clinical specializations acquired through university master’s degrees or continuing education courses. Italian Ministerial Decree 77/2022 reformed territorial healthcare, emphasizing the importance of roles such as that of the healthcare assistant in ensuring the coordination and continuity of care. This decree supports a patient-centered approach that emphasizes multidisciplinary collaboration—core principles that are also reflected in the role of the CM. Given the healthcare assistant’s expertise in managing complex chronic patients and promoting preventive care, this professional is well positioned to act as a CM in specialized healthcare settings and units dedicated to the management of complex diseases.

### 4.1. Case Manager Integration in the Cystic Fibrosis Unit at Careggi University Hospital

The survey results show a consistently high level of agreement among the study participants about the potential benefits of introducing the CM role into the clinical management of CF patients.

The survey results reveal a positive and unanimous consensus regarding the integration of the case manager into the healthcare team of the CF Unit at Careggi University Hospital. Specifically, the majority of respondents selected either “agree” or “strongly agree” in all 12 statements. This finding is further supported by the overall average response, which indicates strong support for the inclusion of a CM in the unit.

These results suggest that the integration of the CM is perceived as beneficial and contributes to improved team dynamics and quality of care. In line with the existing literature, the CM plays a pivotal role in coordinating care, facilitating communication between the multidisciplinary team members, and ensuring a more personalized and continuous approach for patients with complex chronic conditions, such as CF. This can lead to improvements not only in clinical outcomes but also in healthcare workers’ and patients’ satisfaction.

However, an important aspect to consider is the small standard deviation which points to a high degree of consistency in the respondents’ opinions. This may indicate a pre-existing organizational culture that is well-suited to working in multidisciplinary teams or previous positive experience with similar coordination roles.

Although no question was answered with “disagree”, it is important to emphasize that the question on improving the efficiency of the CM in tasks (Q11) received the highest number of neutral responses (*n* = 6). This could be due to perceived challenges or insufficient organizational preparation for the integration of this new role into clinical practice. The majority of physicians (*n* = 4) responded with “either agree or disagree” to this question, indicating some uncertainty or hesitation, albeit to a lesser extent, about the effectiveness and practicality of the CM in improving task efficiency in the clinical setting. When analyzing the other responses, the number of “either agree or disagree” responses was four or less, with no significant differences between professional groups.

The CF Unit at Careggi University Hospital works with a well-structured system in which several healthcare professionals work in a coordinated order to respond to the individual needs of each patient. Given the complex and progressive nature of CF, case management plays a crucial role in ensuring comprehensive and continuous care. The CM plays a critical role in various tasks, such as coordinating multidisciplinary visits, managing follow-up blood tests and diagnostic investigations, and ensuring that specialist consultation occurs at appropriate intervals.

One of the main roles of the CM is to facilitate the coordination of individualized treatment plans and to ensure that the interventions of pulmonologists, dietitians, physiotherapists, and other healthcare professionals are well integrated and adapted to the evolving needs of each patient. As CF requires frequent monitoring, the CM plays a central role in the implementation of telemedicine solutions that enable the remote monitoring of patients, symptom management, and adherence to therapy. This approach promotes patient engagement, ensures timely medical intervention, and reduces the risk of severe exacerbations.

In addition to treatment coordination, the CM is also critical to ensuring treatment adherence, especially as CF patients rely on life-saving medications that are closely monitored by the Italian Drug Agency (Agenzia Italiana del Farmaco—AIFA). These treatments require regular and specific testing to ensure their efficacy and safety. The CM ensures that these tests are carried out promptly and that patients adhere to their prescribed therapies. This prevents treatment delays that could have a significant impact on patient outcomes.

In addition, case management helps optimize healthcare resources by streamlining hospital visits, facilitating insurance authorizations, and minimizing unnecessary diagnostic procedures. The presence of a CM helps reduce inefficiencies within the healthcare system, improving the overall efficiency and cost-effectiveness of CF care.

In addition to these core tasks, the CM takes on new tasks to further improve patient care. One of these tasks is to train patients to use the Electronic Health Record (EHR), a valuable tool that allows patients to access medical test results, view specialist consultations, manage prescriptions, and make appointments. Empowering patients with digital health literacy encourages greater engagement and autonomy, ultimately improving continuity of care. Another important function of the CM is to reduce missed appointments, a common problem in CF care that negatively impacts both patient outcomes and the efficiency of the healthcare system. Missed appointments lead to delays in treatment and a waste of resources. By actively managing appointments and reminding patients of upcoming visits or tests, the CM can mitigate this problem and ensure that valuable time slots are used effectively.

An added benefit of a healthcare professional in the role of CM is the ability to offer telephone advice and face-to-face support when required. This service fulfills several objectives, including the following:

- Health education to encourage better lifestyle habits;
- Monitoring adherence to treatment and reminders for visits or instrumental examinations;
- It acts as a link between patients and various healthcare professionals inside and outside the hospital;
- Such personalized care strengthens the link between patients and their care teams, helping them manage their disease more effectively while improving their physical and psychological well-being.

### 4.2. Leveraging Telemedicine and Educational Support

Another important advantage of integrating the role of the CM into the CF Unit at Careggi University Hospital is the potential use of telemedicine to improve access to care and enable remote monitoring. The CM could use telemedicine tools to monitor the patient’s progress remotely, reducing the need for frequent in-person hospital visits unless absolutely necessary [17]. A literature review aiming to show that telemedicine offers several advantages in the management of patients with CF was conducted [18]. Telemedicine can improve adherence to daily treatments, including respiratory physiotherapy and exercise, facilitate the early detection of pulmonary exacerbations, and address psychological issues. Studies have also shown that activating a telemedicine system for CF allows patients to monitor certain biometric parameters, such as oxygen saturation, weight, and respiratory function, at home and send them directly to the reference center [19]. This would not only reduce the logistical burden for both patients and healthcare providers but also improve the quality of life for patients. Telemedicine has been shown to maintain the same high quality of care, while offering greater convenience for patients with complex chronic conditions such as CF [20]. On the other hand, the main drawback is that no physical examination can be performed, and the lack of face-to-face contact added to technological limits may discourage patients from addressing sensitive issues.

However, the continuity of care; decrease in failures in the fragmentation of the care process; and better communication between the multiplicity of care providers, services, units and level of care could enhance the health of people with complex diseases, valuing humanization and empowering the patients’ sense of control.

The benefits of telemedicine extend across multiple levels. From an environmental perspective, fewer in-person visits means less travel, which in turn reduces CO_2_ emissions, especially for patients who have a good therapeutic response to CFTR modulators and can be effectively monitored remotely [21]. Telemedicine also supports infection control management within clinical settings. By allowing for more flexible appointment scheduling, the CM can ensure that visits are no longer dependent in the type of bacterial colonization in the patient, thus optimizing the clinic’s agenda and reducing the risk of cross-infection among patients.

In addition, the integration of telemedicine could significantly improve the quality of life for CF patients by helping them to better balance personal, professional, and medical responsibilities. This is particularly important for patients living outside the Tuscany region who would otherwise find it more difficult to attend regular hospital visits. The ability to manage follow-up and therapy adherence remotely could be a game changer for these patients, minimizing disruption to their daily lives [22].

Moreover, telemedicine opens up new possibilities for promoting community engagement among CF patients who cannot normally meet in closed spaces due to the risk of cross-infection. Remote group sessions, facilitated by the CM, could be organized for educational and health promotion purposes. These virtual meetings would offer a safe and supportive environment for patients to share experiences, receive counseling, and reinforce self-management strategies, all while minimizing health risks [18].

The integration of a CM into the care pathway for complex chronic disease, such as CF, within the CF Unit of Careggi University Hospital addresses the urgent need for improving coordination and streamlining care. By managing multidisciplinary visits, enhancing adherence to treatment, and using telemedicine, the CM offers significant benefits to both patients and healthcare professionals. Furthermore, the overlap between the competencies of healthcare assistants and CMs suggests that the development of this role could further strengthen the patient-centered healthcare model advocated by Ministerial Decree 77/2022. This model emphasizes the importance of a coordinated multidisciplinary approach to managing chronic conditions, and the CM is well-positioned to play a central role in this evolution.

#### Limitation

Finally, it is important to consider the limitations of the survey itself, such as the number and composition of participants, as these factors could affect the generalizability of the results. For example, if the sample of respondents was limited to a specific group of professionals (e.g., physicians, nurses), the results might reflect the perspective of a single professional group rather than the whole healthcare team.

For the same reason, no bivariate analysis was conducted because the sample size was considered too small to generate robust statistical evidence. The study was designed primarily as an exploratory investigation, aiming to gather preliminary insights rather than to establish statistical associations. Future research with a larger sample may allow for more in-depth statistical analyses, including bivariate and multivariate approaches, to further explore the relationships between variables.

### 4.3. Work in Progress

Following the introduction of the CM role into the clinical practice of the CF Unit, the next step will be a trial period of monitoring, which will be carried out to identify potential areas for improvement. In addition, a study will be conducted with the patients cared for by the CM to determine their satisfaction and the psychological impact of this new role using specific questionnaires. In the first phase of implementation, the focus will be on the management of the most complex patients, with the aim of gradually extending the CM’s support to all patients in the CF Unit of Careggi University Hospital.

## 5. Conclusions

The implementation of a CM in the CF Unit at Careggi University Hospital represents a significant step forward in the management of complex chronic diseases. The results of the survey show a strong consensus among healthcare professionals about the potential benefits of this function, particularly in improving coordination, communication, and patient involvement. By monitoring multidisciplinary care, ensuring adherence to treatment protocols, and using telemedicine, the CM can significantly improve clinical outcomes and patient satisfaction. Furthermore, this approach is in line with the patient-centered healthcare model promoted by recent Italian reforms, which emphasize the importance of collaborative and comprehensive care and incorporate the potential role of the healthcare assistant as a CM in clinical practice. As implementation progresses, continuous evaluation and feedback from patients and healthcare teams will be essential to refine the role of CMs and maximize their impact on patients’ health and quality of life.

## Figures and Tables

**Table 1:** Key elements investigated in the Likert scale questions included in the survey.

Key Elements	Likert Scale-Questions
Understanding of CM’s role	Q1–Q2
Perspective on the implementation of CM in their context	Q3–Q4
Knowledge of CM supportive functions	Q5–Q6
CM role in interdisciplinary collaboration	Q7–Q8–Q9
Opinion about the useful and helpful role of CM	Q10–Q11–Q12

CM: Case Manager.

**Table 2:** Sample of healthcare professionals interviewed using the electronic survey.

Participants	Male/Female	Response Rate
34	13/21	100%

**Table 3:** Distribution of the sample by years of service at Careggi University Hospital.

Years of Service at Careggi University Hospital	N	%
Less than 2 years	2	5.88
2–5 years	21	61.77
6–10 years	2	5.88
11–15 years	3	8.82
16–20 years	2	5.88
Over 20 years	4	11.77

**Table 4:** Distribution of the sample by years of service at the CF Unit.

Years of Service in CF Unit	N	%
Less than 2 years	22	64.71
2–5 years	7	20.59
6–10 years	3	8.82
11–15 years	2	5.88

## Data Availability

Data is contained within the article and Supplementary Materials.

## Supplementary Materials

The following supporting information can be downloaded at: https://www.mdpi.com/article/10.3390/healthcare13090965/s1, File S1: Electronic anonymous questionnaire.
