# Supplementary material for: Integrating Case Management in Cystic Fibrosis Units: A Key to Enhancing Patient-Centered Care
Source: Healthcare (Basel). 2025 Apr 22;13(9):965. doi: 10.3390/healthcare13090965 (PMC12071318; doi:10.3390/healthcare13090965)
Supplement: Supplementary file 1 [file healthcare-13-00965-s001.zip › healthcare-3542869-supplementary.pdf]

Azienda Ospedaliero Universitaria Careggi

QUESTIONNAIRE

What is your professional profile?

Physician

Physiotherapist

Nurse

Health Assistant

Psychologist

What is your gender?

Male

Female

Other

How long have you been working at Careggi University Hospital?

Less than 2 years

2 to 5 years

6 to 10 years

11 to 15 years

16 to 20 years

Over 20 years

How long have you been working in the cystic fibrosis unit?

Less than 2 years

2 to 5 years

6 to 10 years

11 to 15 years

16 to 20 years

Over 20 years

1. I have a clear understanding of the tasks the case manager would perform within our team.

Strongly Disagree	Disagree	Neither disagree not agree	Agree	Strongly Agree
1	2	3	4	5

2. I have a clear understanding of the potential benefits the case manager would bring to our team.

Strongly Disagree	Disagree	Neither disagree not agree	Agree	Strongly Agree
1	2	3	4	5

3. I think the case manager can ensure continuity of care for patients within the hospital environment.

Strongly Disagree	Disagree	Neither disagree not agree	Agree	Strongly Agree
1	2	3	4	5

4. I think the case manager can become a reference point in services where the multidisciplinary team works in shifts.

Strongly Disagree	Disagree	Neither disagree not agree	Agree	Strongly Agree
1	2	3	4	5

5. I think the case manager can act as an intermediary and guide between various professionals in the patient's care pathway.

Strongly Disagree	Disagree	Neither disagree not agree	Agree	Strongly Agree
1	2	3	4	5

6. I think the case manager can ensure continuity of care outside the hospital environment by facilitating communication with external structures (territorial facilities, social and home care, community support associations for patients and their families).

Strongly Disagree	Disagree	Neither disagree not agree	Agree	Strongly Agree
1	2	3	4	5

7. I think the case manager can be supportive for administrative and bureaucratic aspects in the patient's care pathway.

Strongly Disagree	Disagree	Neither disagree not agree	Agree	Strongly Agree
1	2	3	4	5

8. I think the support of the case manager (via phone, email, telemedicine) in the extra-hospital environment can improve patient adherence to daily treatments.

Strongly Disagree	Disagree	Neither disagree not agree	Agree	Strongly Agree
1	2	3	4	5

9. I think the educational support of the case manager can be useful in monitoring the symptoms of patients and their families.

Strongly Disagree	Disagree	Neither disagree not agree	Agree	Strongly Agree
1	2	3	4	5

10. I think the role of the case manager could be critical in the time required for tasks.

Strongly Disagree	Disagree	Neither disagree not agree	Agree	Strongly Agree
1	2	3	4	5

11. I think adequate training is needed for the staff to work effectively with the case manager.

Strongly Disagree	Disagree	Neither disagree not agree	Agree	Strongly Agree
1	2	3	4	5

12. I think our team is ready to introduce the case manager role.

Strongly Disagree	Disagree	Neither disagree not agree	Agree	Strongly Agree
1	2	3	4	5
